# Assessment of the Restoration Potential of Forest Vegetation Coverage in the Alxa Desert Region of China

**DOI:** 10.3390/plants13172536

**Published:** 2024-09-09

**Authors:** Yanlin Pan, Dongmeng Zhou, Jianhua Si, Bing Jia

**Affiliations:** 1 College of Desert Management, Inner Mongolia Agricultural University, Hohhot 010018, China; panyanlin0@163.com; 2 Key Laboratory of Eco-Hydrology of Inland River Basin, Northwest Institute of Eco-Environment and Resources, Chinese Academy of Sciences, Lanzhou 730030, China; jianhuas@lzb.ac.cn (J.S.); jiab@lzb.ac.cn (B.J.)

**Keywords:** water balance, vegetation restoration potential, spatiotemporal variation, scenario simulation, Alxa Desert

## Abstract

To scientifically evaluate the sustainability of tree planting and afforestation in the Alxa Desert region, this study, grounded in the principles of water balance within the natural water cycle, employed multi-source remote sensing products and ground-based measurements to construct a quantitative response relationship model. This model links evapotranspiration (ET) with meteorological variables and the Enhanced Vegetation Index (EVI). Furthermore, the study estimated the recovery thresholds and potential of forest and grassland vegetation coverage in the Alxa Desert region under various precipitation scenarios. The findings reveal that ET exhibited an increasing trend in 84.17% of the Alxa Desert region, with a significant increase observed in 61.53% of the area, indicating positive outcomes from the implementation of the Three-North Shelterbelt Forest Program. Notably, however, ET in the southeastern plain region demonstrated a decreasing trend, which is strongly associated with human activities. The response relationship model demonstrated that linear relationship areas constituted 47.52%, while nonlinear relationship areas accounted for 45.51% of the total. The overall model exhibited an R^2^ value of 0.69, indicating a high level of predictive accuracy. Analysis of forest and grassland coverage revealed that, under wet year scenarios, the vegetation coverage showed a significant trend of recovery, with an average recovery threshold of (75.4 ± 12.5)% and an average recovery potential of (8.5 ± 3.6)%. It is noteworthy that the vegetation coverage in 31.25% of the area had already surpassed the recovery threshold. The outcomes of this study provide a theoretical foundation for the formulation of more scientifically rigorous ecological restoration strategies in the future.

## 1. Introduction

The Alxa Desert region, serving as the first ecological barrier against wind and sand erosion in northern China, holds immense importance for the overall ecological landscape of the country [1]. As a key area for tree planting and afforestation, by 2022, the Alxa Prefecture had accomplished a cumulative task of 5.96 million hectares of tree planting and afforestation. The implementation of these afforestation tasks has significantly improved the ecological environment in the region, with a gradual reduction in desertified land area and a marked increase in vegetation coverage [2]. However, the region is plagued by extreme water scarcity, and precipitation serves as the sole source of water replenishment for artificial vegetation. Soil moisture, replenished by precipitation and subsequently consumed by artificial vegetation, becomes a crucial link in the interrelationship between artificial vegetation, soil, and precipitation [3]. Shallow soil moisture often evaporates rapidly, making it inadequate for supporting vegetation growth. Additionally, the deep groundwater level in the region is difficult for vegetation to directly absorb and utilize, leading to intensified competition for soil moisture among vegetation and subsequent decline or death of artificial vegetation in some areas [4]. Furthermore, ecological restoration in the Alxa Desert region faces uncertainties brought by climate change. Global warming has led to frequent extreme climate events, intensified droughts, and altered precipitation patterns, further complicating ecological restoration efforts. In a state of long-term soil moisture deficit, vegetation ecosystems are prone to degradation, which can weaken their functions of windbreak, sand fixation, and soil and water conservation, potentially triggering a new wave of desertification expansion [5]. Therefore, proposing vegetation restoration thresholds tailored to the characteristics of the Alxa Desert region is of paramount scientific and practical significance for guiding ecological construction, scientifically planning soil and water conservation measures, and predicting future desertification trends in the region.

Vegetation restoration potential, as a quantitative indicator of the gap between vegetation restoration thresholds and current vegetation status, relies heavily on accurate calculations of vegetation restoration thresholds for its assessment [6]. Wang and Shao (2012) [7] estimated the vegetation biomass threshold range of alfalfa in the Mizhi area of northern Shaanxi to be between 2600 and 3500 kg/ha using a water balance model. Fu et al. (2012) [8] determined the leaf area index (LAI) thresholds for *Caragana korshinskii* and *Salix psammophila* in the Liudaogou watershed to be 1.27 and 0.7, respectively, using the SWCCV model. Jia et al. (2019) [9] employed the SHAW model to simulate biomass thresholds of *Alfalfa* and *Caragana Korshinskii* in the Shenmu Liudaogou watershed at 1980 kg/ha and 5050 kg/ha, respectively. These studies primarily focused on the restoration thresholds of typical shrub and grass species in small-scale plots or small watersheds in the Loess Plateau region, making it difficult to comprehensively reflect the spatial variability of vegetation restoration thresholds across the entire Yellow River basin. The rapid development of remote sensing technology has provided new avenues for determining vegetation restoration thresholds at a regional scale. Gao et al. (2017) [10] extracted vegetation restoration thresholds for different habitat patches using the histogram statistics method based on the “similar habitat principle”. To address the spatial heterogeneity of habitat condition classification standards, Zhang et al. (2020) [11] proposed an improved calculation method for “vegetation restoration thresholds based on sliding window similar habitats”. While such methods are simple and feasible, they struggle to fully consider the impact of micro-topographic changes on habitat conditions when delineating “similar habitat” patches. Moreover, the extracted thresholds merely represent the maximum vegetation parameter values within patches during the study period, rather than the vegetation thresholds that maintain the balance between vegetation water consumption and rainfall supply.

Furthermore, Feng et al. (2016) [12] constructed a quantitative response relationship between evapotranspiration (ET) and gross primary productivity (GPP) in the Loess Plateau ecosystem and simulated GPP thresholds under different ecosystem water supply scenarios. Liang et al. (2019) [13] further considered the influence of site condition heterogeneity on the quantitative response relationship between ET and GPP. However, GPP, as an indicator representing the carbon fixation capacity of vegetation photosynthesis (gC·m^−2^·a^−1^), despite its close biophysical association with ET, is difficult to directly apply in ecological construction engineering practices. Zhang et al. (2018) [14] used the Eagleson eco-hydrological model and ecological optimality theory to simulate the restoration thresholds and spatial distribution of vegetation coverage in the Loess Plateau under different climate scenarios. However, the calculation process of the eco-hydrological model is complex, and accurate acquisition of soil and vegetation parameters in the model is challenging, introducing significant uncertainty into the simulation accuracy.

Therefore, this study utilizes existing remote sensing products of ET and vegetation parameters, considering the linear and nonlinear response relationships between ET and vegetation as well as the interactive effects between vegetation and meteorological factors. By employing a stepwise regression method to construct the optimal response relationship between ET, meteorological factors, and vegetation indices pixel by pixel, based on the principle of water balance, this study calculates the restoration thresholds of forest and grassland vegetation coverage under different climatic conditions. It aims to provide a theoretical basis for ecological construction in the Alxa Desert region.

## 2. Materials and Methods

### 2.1. Study Area Description

**Figure 1:**
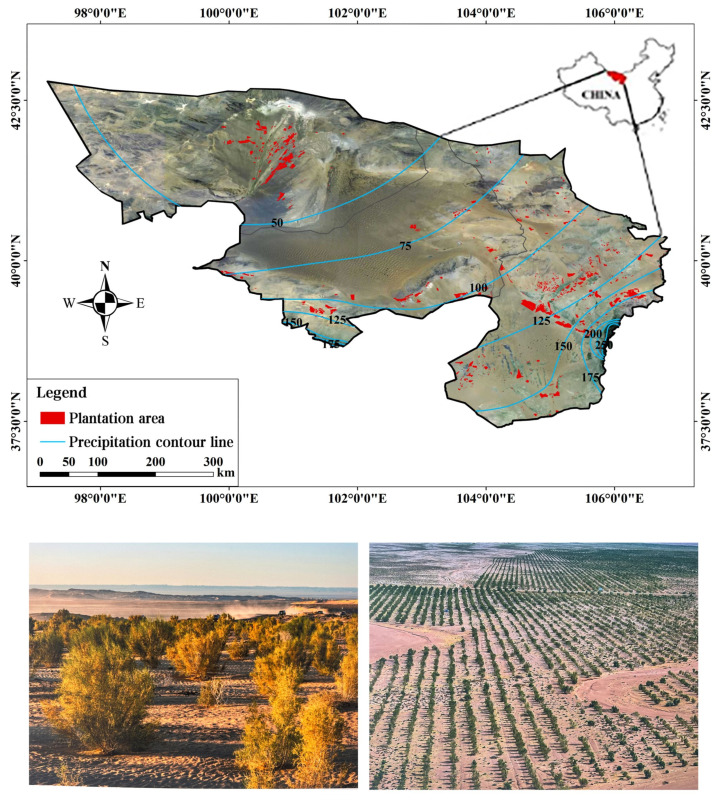
Location of the study area in the Alxa Legue desert, China.

The research area is located in Alxa League, Inner Mongolia Autonomous Region (37°24′42°47′ N, 97°10′106°53′ E) (Figure 1). The climate of this region is characterized by extreme aridity and scarce precipitation, with an annual average precipitation of only 40–150 mm, while the evaporation rate far exceeds the precipitation, exceeding 2000 mm annually. To prevent further desertification and improve the local ecological environment, by 2022, Alxa League had completed a total of 5.96 million hectares of tree planting tasks. The main tree species for afforestation include *Haloxylon ammodendron*, *Populus euphratica*, *Hippophae rhamnoides* L., *Hedysarum scoparium*, and *Calligonum mongolicum* T. Through the implementation of tree planting tasks, the ecological environment of the region has been significantly improved, with the area of desertified land decreasing annually and vegetation coverage increasing markedly. However, due to the arid climate and strong evaporation in this region, artificial vegetation has been subject to severe drought stress over time, leading to the decline and death of vegetation in some areas. Therefore, as a region that has implemented large-scale tree planting under extreme climate and water-scarce conditions, defining the threshold of artificial vegetation coverage under the constraints of climate–soil moisture–vegetation carrying capacity in the Alxa region holds significant research value and practical implications for ecological restoration and stable vegetation maintenance in similar arid regions.

### 2.2. Research Methods

#### 2.2.1. Evaluation Method for ET Product Accuracy

The spatial average of ET products within a 3 × 3 pixel grid (1.5 km × 1.5 km) adjacent to the coordinates of the small meteorological station was extracted. The accuracy of PML_V2 ET and MOD16 A2 GF ET products was assessed using the Root Mean Square Error (RMSE), Mean Relative Error (MRE), and Nash Efficiency Coefficient (Nash) indicators.

(1) $$ET_{WB_{i}}=P_{i}-R_{i}-\Delta S_{i}$$

where $ET_{WB_{i}}$ represents the evapotranspiration (mm) in the i-th year interval; $P_{i}$ represents the measured rainfall (mm) in the *i*-th year interval; $R_{i}$ represents the measured runoff (mm) in the *i*-th year interval; and $\Delta S_{i}$ represents the interval TWSC (mm) retrieved by the GRACE gravity satellite in the *i*-th year.

**Figure 2:**
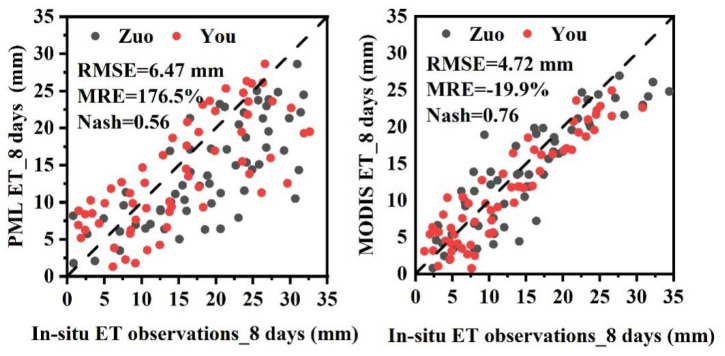
Validation Results of MODIS and PML_V2 ET Products Based on Station Observation Data.

This study selected the most widely used MODIS ET (http://modis.gsfc.nasa.gov/) product and the improved PML_V2 ET (https://code.earthengine.google.com/37010f349cdfbb50a46eea0d5f7d1db6) product for accuracy evaluation. The validation results are shown in Figure 2. Figure 2 illustrates the validation accuracy of MODIS and PML_V2 ET products at two flux sites (Zuo and You). As can be seen from the figure, the root mean square error (RMSE) of the PML_V2 ET product at the site scale is 6.47 mm, the mean relative error (MRE) is 176.5%, and the Nash efficiency coefficient (Nash) is 0.56; while the RMSE, MRE, and Nash coefficient of the MODIS ET product are 4.72 mm, −19.9%, and 0.76, respectively. The validation results indicate that the RMSE and MRE of the PML_V2 ET product are both smaller than those of the MODIS ET product, indicating relatively smaller model simulation errors; moreover, the Nash coefficient is higher than that of the PML_V2 ET product, demonstrating higher simulation accuracy in the Alxa Desert region and the ability to accurately reflect the spatiotemporal dynamic changes of ET in this area.

#### 2.2.2. Trend Slope Analysis Method

The linear slope method and F-test were used to calculate the ET trend and its significance level in the Loess Plateau from 2000 to 2020. The formula for calculating the linear slope is as follows [15]:

(2) $$\mathrm{Slope}=\frac{n\times \sum_{i=1}^{n}\;\;\left(i\times X_{i}\right)-\sum_{i=1}^{n}\;\;i\sum_{i=1}^{n}\;\;X_{i}}{n\times \sum_{i=1}^{n}\;\;i^{2}-{\left(\sum_{i=1}^{n}\;\;i\right)}^{2}}$$

where $X_{i}$ represents the ET value in the i-th year; *n* represents the total number of years. When Slope > 0, ET shows an increasing trend; when Slope < 0, ET shows a decreasing trend. When the significance level of the F-test for the ET trend is *p* < 0.05, ET shows a significant change.

#### 2.2.3. Construction of Response Relationships between ET and Meteorological Elements and EVI

Drawing on previous research results and experience, this study will construct a quantitative response relationship between ET and rainfall, temperature, sunshine duration, saturated vapor pressure difference, and EVI. The Enhanced Vegetation Index (EVI) data were obtained from the MODerate Resolution Imaging Spectroradiometer (MODIS) product, specifically the MOD13Q1 dataset. This dataset provides a 16-day composite image at a spatial resolution of 250 m, which was chosen for its high temporal and spatial resolution, as well as its ability to capture the dynamic changes in vegetation conditions. The EVI data were processed using the MODIS Reprojection Tool (MRT) to ensure consistency with the spatial resolution and projection of the other meteorological datasets used in this study. In the process of constructing the response relationship, the nonlinear relationship between vegetation and ET and the interaction between meteorological elements and vegetation are fully considered. The specific formula is as follows [16]:

(3) $$\begin{array}{c} ET=\beta_{0}+\beta_{EVI}\times EVI+\beta_{i}\times X_{i}+\varepsilon \\ X_{i}=\left\{{EVI}^{2},C_{j},C_{j}\times EVI\right\},j=1,2,3,4 \end{array}$$

In the formula, ET represents actual evapotranspiration; $\beta_{EVI}$ represents the sensitivity coefficient of ET to EVI; $X_{i}$ represents the subset of other variables; $\beta_{i}$ represents the sensitivity coefficient of each variable in the selected variable subset $X_{i}$. In the multiple linear equation where $X_{i}$ includes EVI^2^, it characterizes the vegetation; $C_{j}$ characterizes the linear effects of four meteorological elements (rainfall, temperature, sunshine duration, and saturated vapor pressure difference); $C_{j}$× EVI characterizes the interaction between meteorological elements and vegetation. This study uses the multiple stepwise regression method to select the best variable subset $X_{i}$ for each grid cell, thereby establishing the “optimal” quantitative response relationship between ET and meteorological factors and EVI in each grid cell.

To investigate the multicollinearity present in the data of this study, Variance Inflation Factor (VIF) analysis was employed. The core idea is to calculate the correlation of each feature with other features and use the VIF value to indicate the degree of correlation for each feature. The calculation method of VIF involves treating each feature as the dependent variable and the remaining features as independent variables, fitting a linear regression model, and then calculating the ratio of the mean square error of the independent variables to that of the dependent variable, specifically:

(4) $$R_{i}^{2}=\frac{\sum_{j=1}^{n}\;{\left({\hat{p}}_{ij}-{\bar{p}}_{ij}\right)}^{2}}{\sum_{j=1}^{n}\;{\left(p_{ij}-{\bar{p}}_{ij}\right)}^{2}}\;$$

(5) $$VIF_{i}=\frac{1}{1-R_{i}^{2}}\;$$

In the formula, $R_{i}^{2}$ represents the coefficient of determination for indicator variable i; $VIF_{i}$ represents the Variance Inflation Factor for indicator variable i; ${\hat{P}}_{ij}$ represents the estimated value of indicator variable i for region j; and ${\bar{P}}_{ij}$ represents the mean value of indicator variable i. When 0<VIF<10, it indicates no multicollinearity; when 10<VIF<100, it suggests the presence of strong multicollinearity; and when VIF>100, it indicates severe multicollinearity.

To verify that this model can effectively handle multicollinearity among data, this study analyzed the multicollinearity between EVI and $C_{j}$ × EVI, and employed the Variance Inflation Factor (VIF) test to evaluate the extent of multicollinearity. The results of the VIF collinearity test are presented in Table 1. As indicated in Table 1, by calculating the VIF values of each variable, it was found that the VIF value for EVI is 1.17, the value for $C_{j}$ × EVI is 1.09, and the value for EVI^2^ is 1.23, indicating that each explanatory variable provides unique information and does not negatively impact prediction accuracy. Therefore, the model constructed in this study is suitable for further analysis.

#### 2.2.4. Classification of Wet, Normal, and Dry Years

Based on precipitation data from 1960 to 2018, the P-III frequency distribution curve was used to analyze the precipitation frequency of each pixel. According to the classification method of [17], years with a precipitation frequency of less than 25% were classified as wet years; years with a precipitation frequency between 25% and 75% were classified as normal years; years with a precipitation frequency greater than 75% were classified as dry years. The probability density function of the P-III frequency distribution curve is as follows:

(6) $$f(x)=\frac{\beta^{x}}{\Gamma (\alpha )}{\left(x-a_{0}\right)}^{\alpha -1}e^{-\beta \left(x-a_{0}\right)}$$

where Γ(α) is the gamma function; α, β, and a_0 are the shape parameter, scale parameter, and location parameter of the P-III frequency distribution, respectively, and α > 0, β > 0.

#### 2.2.5. Estimation of Vegetation Cover Recovery Potential under Different Scenarios

According to the principle of water balance, evapotranspiration is the remaining part after deducting runoff and terrestrial water storage changes from rainfall. When rainfall replenishment can meet the water consumption demands of the ecosystem, the succession of vegetation communities is sustainable. Under the condition of maintaining the balance between rainfall replenishment and ecosystem ET, the maximum value of ET is the precipitation, i.e., there is no runoff or change in water storage [18].

(7) ET=P−R−ΔS

where ET represents evapotranspiration; P represents precipitation; R represents runoff; ΔS represents terrestrial water storage changes. Substituting $ET_{max}=P$ into Formula (7) to calculate the EVI value, which is the EVI threshold that can maintain the balance between rainfall supply and ecosystem water consumption. Combining surface cover product data, the pixel dichotomy method is used to convert the EVI threshold into forest and grassland vegetation cover [19].

(8) $${FVC}_{i,j}=\frac{{EVI}_{i,j}-{EVI}_{\min}}{{EVI}_{\max,j}-{EVI}_{\min}}$$

where ${FVC}_{i,j}$ represents the vegetation cover of the *i*-th pixel in the *j*-th type of forest and grassland vegetation; ${EVI}_{i,j}$ represents the EVI value of the *i*-th pixel in the *j*-th type of forest and grassland vegetation; ${EVI}_{max,j}$ represents the EVI value corresponding to the 95% quantile of the annual maximum statistical histogram for the *j*-th type of forest and grassland vegetation; ${EVI}_{min}$ represents the EVI value corresponding to the 5% quantile of the annual minimum statistical histogram for bare land types.

Based on the principle of water balance, evapotranspiration (ET) represents the remaining component after accounting for runoff (R) and terrestrial water storage changes (ΔS) from rainfall. Sustainable vegetation community succession occurs when rainfall replenishment meets ecosystem water consumption demands. To determine the threshold for vegetation cover recovery, we identify the Enhanced Vegetation Index (EVI) threshold that maintains balance between rainfall replenishment and ecosystem ET. We set the $ET_{max}$ equal to precipitation (P), indicating balance without runoff or water storage changes. Substituting this into the ET calculation Formula (7) solves for the corresponding EVI threshold. Since the EVI threshold varies with vegetation types, we combine surface cover product data and use the pixel dichotomy method to convert it into vegetation cover thresholds for specific types like forests and grasslands, considering their EVI contributions and ecosystem proportions. We determine vegetation cover by calculating EVI differences relative to minimum and maximum values for each vegetation type, with maxima at the 95th percentile of annual maximums and minima at the 5th percentile of annual minimums for bare land. This yields a vegetation cover recovery threshold (FVC_thr_) reflecting optimal cover sustained by available water under current climate. The difference between FVC_thr_ and current vegetation cover (FVC_cur_) represents recovery potential (FVC_pot_), indicating potential vegetation growth under water balance conditions. The difference between the vegetation cover recovery threshold and the current vegetation cover status is the vegetation cover recovery potential, calculated as follows:

(9) $${FVC}_{\mathrm{pot}}={FVC}_{thr}-{FVC}_{\mathrm{cur}}$$

where ${FVC}_{pot}$ represents the vegetation cover recovery potential; ${FVC}_{thr}$ represents the vegetation cover recovery threshold; ${FVC}_{cur}$ represents the current vegetation cover status.

## 3. Results and Analysis

### 3.1. Spatiotemporal Characteristics of ET and EVI in the Alxa Desert Region

**Figure 3:**
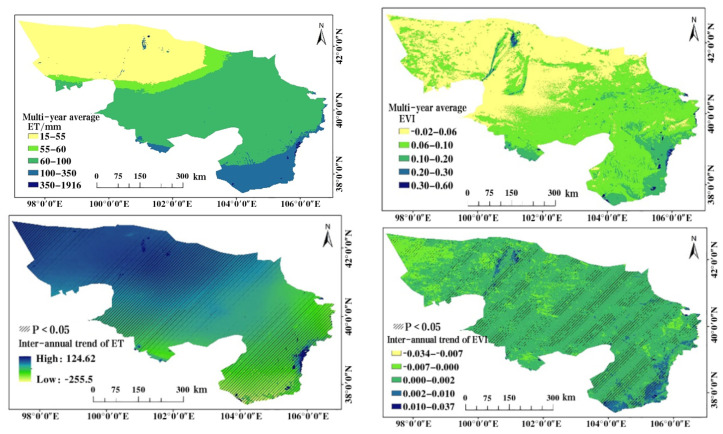
2000–2020 Spatiotemporal Distribution of ET and EVI in the Alxa Desert Region.

The regions showing an increasing trend in evapotranspiration (ET) in the Alxa Desert Region account for 84.17% of the total area (Figure 3). Notably, 61.53% of the regions exhibit a significant increase in ET, primarily concentrated in the central and western parts of the Alxa Desert Region, which are key implementation areas of the Three-North Shelterbelt Forest Program (a major ecological construction project aimed at improving the ecological environment in northern China). This indicates that the implementation of the Three-North Shelterbelt Forest Program in these areas has achieved positive results and has had a beneficial impact on the local ecological environment. However, in the southeastern plain area of the Alxa Desert Region, a decreasing trend in ET is observed, accounting for 15.83% of the area. This decrease can be attributed to the urbanization process and other intense human activities in the region, such as overgrazing, which reflect the potential negative impacts of human activities on the natural environment”.

**Figure 4:**
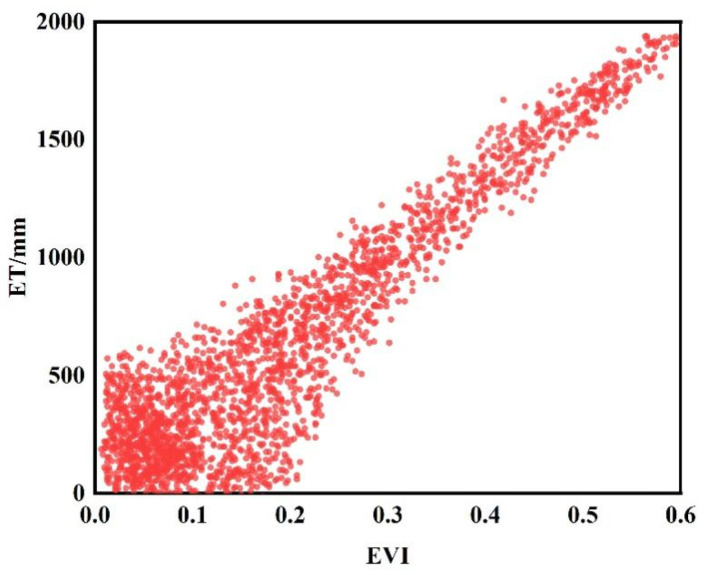
Scatter Plot of EVI and ET Relationship in the Study Area.

Meanwhile, the Enhanced Vegetation Index (EVI) in the Alxa Desert Region has also shown a positive growth trend, with an area accounting for 92.12% exhibiting an increasing trend. Among these areas, 85.56% show a significant increase in EVI. Nevertheless, it is important to note that in the southeastern plain area and some scattered regions of the Alxa Desert Region, EVI has experienced a significant decreasing trend, although these areas only account for 2.25% of the total area. Further analysis reveals that the annual growth rate of ET for forest and grassland vegetation reaches 4.13 mm·year^−1^. For the forest and grassland vegetation in the Alxa Desert Region, the annual growth rate of EVI is 0.0027/year, indicating a slow but steady growth trend. Through the addition of a scatter plot, the relationship between Evapotranspiration (ET) and the Enhanced Vegetation Index (EVI) was further analyzed. Figure 4 reveals a clear positive correlation between ET values and EVI values within the study area, indicating that an increase in ET may be associated with an increase in EVI. This finding supports the viewpoint that active vegetation metabolism could lead to concurrent increases in both ET and EVI. Specifically, as vegetation grows and metabolic activity intensifies, the utilization of water and transpiration by vegetation also increase, resulting in elevated ET. Concurrently, vegetation growth and lushness contribute to an increase in EVI values. Therefore, it can be concluded that the increase in ET is correlated with the growth in EVI within the study area, further confirming the significant impact of vegetation ecological conditions on the hydrological cycle and ecological environment.

### 3.2. Spatial Distribution Characteristics of the Response Relationship between ET and EVI

Using a stepwise multiple regression method, this study constructed response relationship models between evapotranspiration (ET) and meteorological factors as well as EVI for each pixel individually. These response relationships can be mainly classified into three forms: (1) Only EVI is related to ET, indicating that in some regions, changes in evapotranspiration are primarily determined by vegetation conditions; (2) Both EVI and the square of EVI (EVI^2^) are related to ET, reflecting a possible nonlinear relationship between ET and EVI; (3) The product of EVI and a specific meteorological factor C_j_ (EVI × C_j_) is related to ET, suggesting that ET is not only influenced by vegetation conditions but also controlled by specific meteorological factors.

**Figure 5:**
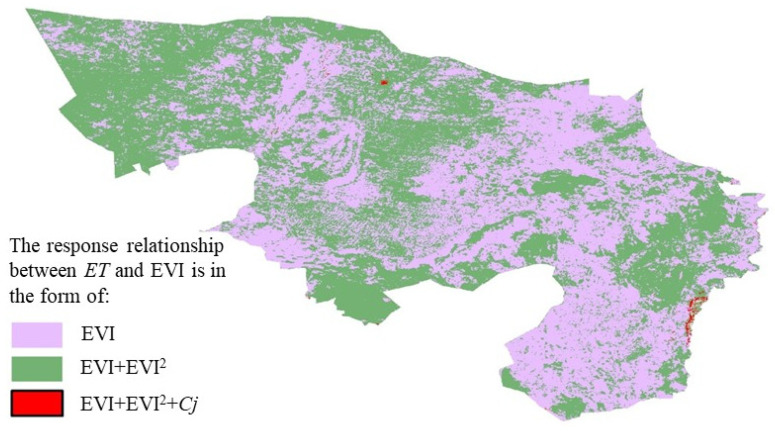
Response relationship between ET and EVI.

The spatial distribution of different response relationship forms is shown in Figure 5. Statistical results indicate that the area where ET has a linear relationship with EVI accounts for 47.52% of the total. In comparison, the area where ET has a nonlinear relationship with EVI accounts for 45.51% of the total, and within these nonlinear relationship areas, 6.97% are also influenced by the interaction between EVI and meteorological factors. Overall, the R^2^ value of the multiple regression model is 0.69, indicating that the model performs well. Specifically, the R^2^ values for the three response relationship models—where only EVI is related to ET, both EVI and EVI2 are related to ET, and both EVI and EVI × Cj are related to ET—are 0.71, 0.74, and 0.62, respectively. The relatively lower R^2^ value of 0.62 for the model including both EVI and EVI × C_j_ may be attributed to the increased complexity introduced by the interaction term between EVI and meteorological factors, as well as the inherent variability of these factors. Despite this, the model still demonstrates good explanatory power under different response relationships. It is noteworthy that the response relationship models constructed for 75.32% of the regions are significant (as shown in Figure 5), further proving the validity of the model. The average root mean square error (RMSE) of the response relationship models constructed in this study is 25.3 mm, which is relatively low, indicating high accuracy of model predictions. To more comprehensively assess the uncertainty in ET product validation, this study further calculated the average RMSE of ET simulated values from the multiple regression model as 49.5 mm using the error propagation law. This result provides a quantitative assessment of model prediction error, helping us to gain a deeper understanding of the model’s performance and application potential under different conditions.

### 3.3. Recovery Potential of Forest and Grassland Vegetation Cover under Scenarios of High, Normal, and Low Water Years

**Figure 6:**
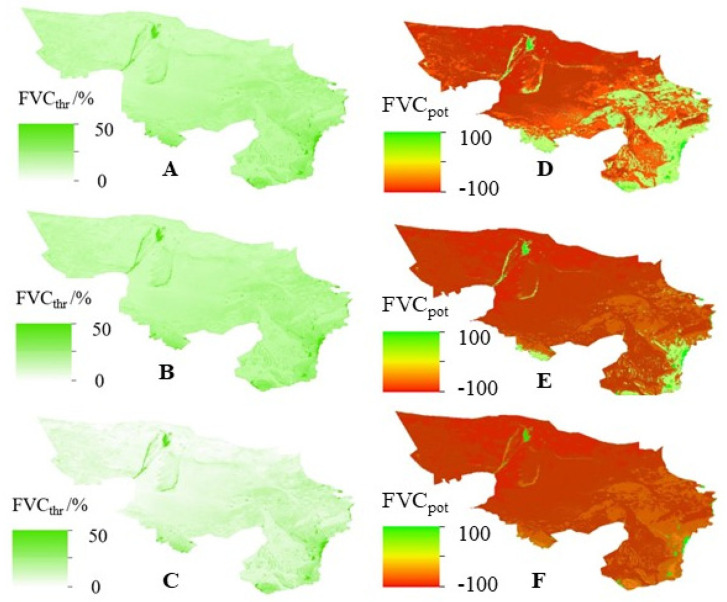
Recovery Threshold and Potential of Forest and Grass Vegetation Coverage, (**A**) FVC_thr_ in Wet Years; (**B**) FVC_thr_ in Normal Years; (**C**) FVC_thr_ in Dry Years; (**D**) FVC_pot_ in Wet Years; (**E**) FVC_pot_ in Normal Years; (**F**) FVC_pot_ in Dry Years. FVC_thr_: Restoration threshold of fractional vegetation cover; FVC_pot_: Restoration potential of fractional vegetation cover.

Through a deep analysis of the forest and grassland cover in the Alxa Desert region under different scenarios of high, normal, and low water years, Formulas (6)–(9) were used to quantitatively calculate the recovery thresholds and recovery potential of forest and grassland cover under these scenarios. Furthermore, based on the calculation of the root mean square error (RMSE) of ET simulated values mentioned in Section 2.2, we obtained an average RMSE of 5.4% for the recovery thresholds and recovery potential of forest and grassland cover. In the scenario of a high water year, the forest and grassland vegetation cover in Alxa exhibited a significant trend of recovery. Specifically, the average recovery threshold was (75.4 ± 12.5)%, indicating that above this threshold, forest and grassland vegetation has a greater likelihood of achieving self-recovery. At the same time, the average recovery potential was (8.5 ± 3.6)%, suggesting that under current conditions, forest and grassland vegetation still has considerable room for recovery and growth (Figure 6). It is noteworthy that the forest and grassland vegetation cover on 31.25% of the area has already exceeded the recovery threshold, with these areas mainly distributed in the central and western parts of the Alxa Desert region, which are also key implementation areas of the Three-North Shelterbelt Forest Program. However, the forest and grassland vegetation cover on 68.75% of the area has not reached the recovery threshold but has recovery potential, with these areas mainly distributed near the Helan Mountains in eastern Alxa.

## 4. Discussion

### 4.1. Discussion on the Temporal and Spatial Variation Characteristics of ET and EVI in the Alxa Desert Region

Since the implementation of the Three-North Shelterbelt Forest Program, vegetation restoration in the Alxa Desert region has shown remarkable results, with reduced soil erosion and significantly improved ecosystem services [20]. However, soil moisture has emerged as a major limiting factor for vegetation succession in arid and semi-arid regions. Numerous studies based on measured soil moisture data at different depths indicate that excessive consumption of soil moisture by artificial vegetation exceeds local rainfall replenishment, leading to the phenomenon of dry soil layers in deep profiles [20]. To address this, implementing fencing and enclosure for vegetation protection, promoting eco-friendly agricultural practices, and strengthening vegetation conservation efforts are crucial. At the regional scale, a large number of previous studies have shown that vegetation restoration in this area is approaching the threshold of water resource carrying capacity [21,22], which is generally consistent with the qualitative conclusions of this study. This study reveals the temporal and spatial variation characteristics of evapotranspiration (ET) and Enhanced Vegetation Index (EVI) in the Alxa Desert region, finding that ET shows an increasing trend in most areas, particularly significant in the central and western parts where the Three-North Shelterbelt Forest Program is intensively implemented, indicating a positive impact of ecological engineering on the local environment. However, the significant decrease in ET in the southeastern plain area may be closely related to urbanization and other intense human activities, which suggests that we must pay more attention to the potential negative impacts of human activities on the ecological environment and take corresponding protective measures, such as implementing sustainable urban planning, promoting water-saving technologies, and enhancing environmental regulations.

Meanwhile, the positive growth of EVI in the Alxa Desert region also reflects a significant improvement in the region’s ecological conditions, but the decline in EVI in scattered areas cannot be ignored and requires further investigation and research. Although the inter-annual growth rate of ET and EVI for forest and grassland vegetation is slow but stable, indicating that ecological restoration is a long-term and continuous process that requires ongoing monitoring and management, as well as the implementation of the aforementioned protective measures to ensure sustainable ecological development.

### 4.2. The Spatial Distribution Characteristics of ET and EVI Response Relationships

Since the implementation of the Three-North Shelterbelt Forest Program in the Alxa Desert region, vegetation cover has significantly improved, soil erosion has gradually weakened, and various ecosystem service functions have significantly increased. Climate change and human activities jointly drive vegetation changes in this region [23], with precipitation being the main influencing factor for vegetation cover increase, consistent with the findings of Tian et al. (2022) [24]. On the other hand, human activities are also important factors affecting vegetation changes and have a dual impact on EVI [25], such as afforestation significantly increasing vegetation cover on the Loess Plateau, while rapid urbanization leads to a rapid decrease in regional vegetation cover.

This study uses a stepwise multiple regression method to construct response relationship models between evapotranspiration (ET) and meteorological elements as well as the Enhanced Vegetation Index (EVI) for each pixel and finds different response relationship forms in different regions. Linear relationships dominate in most areas, indicating that changes in evapotranspiration are mainly determined by vegetation conditions in these regions. However, nonlinear relationships also account for a certain proportion, reflecting the possible complex relationship between ET and EVI. In addition, the interaction between meteorological elements and EVI has a significant impact on ET in some areas, indicating that ET is not only affected by vegetation conditions but also modulated by specific meteorological elements. The high R^2^ values of the models indicate that they can well explain the relationship between ET and EVI. At the same time, the response relationship models constructed for most areas are significant, and the average RMSE of the models is relatively low, further proving the validity and prediction accuracy of the models. This finding provides a powerful tool for future research on the relationship between ET, vegetation conditions, and meteorological elements, and helps to gain a deeper understanding of the complexity and dynamics of ecosystems. The inversion accuracy of ET in the ecosystem directly determines the accuracy of estimating vegetation restoration thresholds using this method. Due to limited shared data available for collection, there is some uncertainty in the validation accuracy of this study. In the future, more field-measured soil moisture and ET flux data can be collected to validate and improve various ET inversion models.

### 4.3. Potential for Vegetation Cover Recovery of Forests and Grasslands under Scenarios of Wet, Normal, and Dry Years

Previous studies used the “similar habitat principle” [26] and the histogram statistical method to extract vegetation restoration thresholds for different habitat patches, but errors in “similar habitat” patch classification can significantly affect the extraction results, and the calculation process did not consider the balance between rainfall supply and ecosystem ET, so the extraction results may represent the maximum vegetation cover under conditions of excessive soil moisture consumption. This study, from the perspective of the water balance principle, constructs a quantitative response relationship between ecosystem ET and meteorological elements and vegetation indices, and quantitatively calculates the forest and grassland vegetation restoration thresholds that can maintain the balance between rainfall supply and ecosystem water consumption, which can be used for the sustainability evaluation of forest and grassland vegetation. This is similar to the research approach of Wang et al. (2016) [26] in estimating vegetation restoration thresholds, but the previous study was conducted at the county scale, and the vegetation indicator GPP (gC·m^–2^·a^–1^) used is not convenient for direct application in ecological construction engineering practice, while this study calculates the forest and grassland vegetation restoration thresholds within each 500 m × 500 m grid, and the results can be directly used in engineering practice. In addition, previous studies mainly used recent remote sensing observation data to extract vegetation thresholds, which only represent the dynamic changes and restoration thresholds of forest and grassland vegetation under climate conditions in the past ten years, and did not fully consider the impact of different climate scenarios in the future on vegetation thresholds.

This study conducted a deep analysis of the forest and grassland cover restoration thresholds and recovery potential under different hydrological year scenarios in the Alxa Desert region and found that the forest and grassland vegetation cover exhibited a significant recovery trend under high water year scenarios. This finding is important for guiding ecological restoration work in the region. However, the forest and grassland vegetation cover in a large proportion of the area has not reached the restoration threshold but has recovery potential, mainly distributed near the Helan Mountains. This indicates that although the ecological environment in some areas has improved, there are still large areas that require further ecological restoration work. It is worth noting that achieving self-maintenance of the ecological environment is a long-term goal, and its realization depends on various factors such as climate stability, sustained ecological restoration efforts, and the inherent resilience of the ecosystem. Predicting the exact timeline for this is complex and requires further research. At the same time, the average RMSE of the restoration thresholds and recovery potential is low, indicating that the results of this study are reliable and practical. Future attention should continue to be paid to changes in the ecological environment of these areas, and effective ecological restoration measures should be taken to promote vegetation recovery and growth. In addition, further research is needed on the variation patterns of forest and grassland vegetation cover restoration thresholds and recovery potential under different hydrological year scenarios to provide a theoretical basis for formulating more scientific ecological restoration strategies, including the exploration of conditions and timelines for ecological self-maintenance.

## 5. Conclusions

Based on the principle of natural water cycle water balance, this study uses multi-source remote sensing products and measured ground data to construct a quantitative response relationship between ET and meteorological elements and EVI, and quantitatively estimates the restoration thresholds and recovery potential of forest and grassland vegetation cover in the Alxa Desert region under different precipitation scenarios. The main research conclusions are as follows:

(1) Evapotranspiration (ET) in the Alxa Desert region shows an increasing trend in 84.17% of the area, with a significant increase in 61.53% of the area, mainly concentrated in the key implementation areas of the Three-North Shelterbelt Forest Program, indicating that the implementation of the program has achieved positive results. However, ET in the southeastern plain area shows a decreasing trend, which is closely related to human activities such as urbanization.
(2) This study uses a stepwise multiple regression method to construct response relationship models between ET and meteorological elements and EVI, with linear relationship areas accounting for 47.52% and nonlinear relationship areas accounting for 45.51%. The overall model R^2^ value is 0.69, indicating good performance, and 75.32% of the regional models are significant. The average RMSE of the model is 25.3 mm, with high prediction accuracy, and the average RMSE of the ET simulation value is 49.5 mm, providing a quantitative assessment of model prediction error.
(3) Through the analysis of forest and grassland cover under different hydrological year scenarios in the Alxa Desert region, the average RMSE of the restoration thresholds and recovery potential is calculated to be 5.4% using quantitative calculation methods. Under high water year scenarios, the forest and grassland vegetation cover shows a significant recovery trend, with an average restoration threshold of (75.4 ± 12.5)% and an average recovery potential of (8.5 ± 3.6)%. The forest and grassland vegetation cover in 31.25% of the area has exceeded the restoration threshold, mainly distributed in the central and western parts where the Three-North Shelterbelt Forest Program is intensively implemented; while 68.75% of the area has not reached the threshold but has recovery potential, mainly distributed near the Helan Mountains.

This study constructs response relationship models between ET and meteorological elements and EVI and finds different response relationship forms in different regions, and the models have high explanatory power and prediction accuracy, providing a powerful tool for future research. Furthermore, from the perspective of the water balance principle, this study quantitatively calculates the forest and grassland vegetation restoration thresholds that can maintain the balance between rainfall supply and ecosystem water consumption, and the results can be directly used in engineering practice. The study finds that the forest and grassland vegetation cover.

## Figures and Tables

**Table 1:** VIF Collinearity Test.

Variable	VIF	1/VIF
EVI	1.17	0.85
$C_{j}$ × EVI	1.09	0.92
EVI^2^	1.23	0.81

## Data Availability

The data that support the findings of this study are available from the corresponding author on reasonable request.
